# To collar or not to collar. Views of pre-hospital emergency care providers on immobilisation without cervical collars: a focus group study

**DOI:** 10.29045/14784726.2021.6.6.1.38

**Published:** 2021-05-01

**Authors:** Lee Thompson, Gary Shaw, Charlotte Bates, Christopher Hawkins, Graham McClelland, Peter McMeekin

**Affiliations:** North East Ambulance Service NHS Foundation Trust ORCID iD: http://orcid.org/0000-0002-0820-1662; North East Ambulance Service NHS Foundation Trust; Northumbria Specialist Emergency Care Hospital; Sunderland Royal Hospital; North East Ambulance Service NHS Foundation Trust; Northumbria University

**Keywords:** focus group, pre-hospital, semi-rigid collar, spinal cord injury

## Abstract

**Background:**

Spinal cord injury (SCI) is a rare event, with high numbers of patients unnecessarily immobilised with no potential benefit based on limited evidence from the 1950s and 1960s. Contemporary opinion now challenges the notion that traditional immobilisation prevents movement and protects the spine. Current literature suggests that these methods which include semi-rigid collars can potentially cause more movement of the spine and harm the patient. The purpose of this study was to explore the views and perspectives of pre-hospital care providers on immobilising patients without the use of a semi-rigid collar.

**Methods:**

Focus groups were used to allow individuals to discuss and comment on a new method of immobilisation which omits the semi-rigid collar and to capture the thoughts, feelings and experiences of participants. Thematic analysis of the coded transcriptions was used to identify emerging themes.

**Results:**

Three focus groups were conducted with 15 participants in each. Participants were all exposed to patients sustaining trauma within their professional roles. Six intertwined themes emerged from the analysis: communication, conflict, education/training, empowerment, risk and the patient. Woven between these themes are the complex interactions that bring together the inter-professional relationships with other emergency services and hospital staff, the patient, the public and pre-hospital care providers.

**Discussion:**

Existing immobilisation practices are being challenged, with clinicians empowered to tailor practice to meet specific patient needs. There is limited empirical evidence to support current immobilisation practices. Contemporary literature suggests current practices may potentially cause harm. New pragmatic immobilisation practices are gradually being adopted by some pre-hospital care providers.

**Conclusion:**

This study explored the perspectives of pre-hospital care providers on immobilising patients without the use of a semi-rigid collar for potential SCI. The consensus of the participants supports a pragmatic approach to managing potential SCI that provides safe, high-quality patient-centred care.

## Introduction

### Problem formulation

The literature highlights a variable global incidence of traumatic spinal cord injury (SCI) of between 9.2 and 246 per million persons per year, with a prevalence of 236 to 1298 per million population (Furlan et al., 2013). SCI is, however, a very rare event affecting 40,000 people in the UK (All Party Parliamentary Group on Spinal Cord Injury, 2015), with half of SCIs occurring due to fractures in the cervical spine (Spinal Injuries Association, 2009). In their pre-hospital study, Oteir et al. (2016) estimated that of the 106,059 patients identified with potential traumatic SCI, less than 0.5% had confirmed SCI. As a consequence, a high volume of patients are unnecessarily immobilised with no potential benefit.

The colloquially termed ‘collared and boarded’ immobilisation (semi-rigid collar, long board/scoop, blocks and tape/straps) has been standard clinical practice for transporting patients with potential spinal injuries for many years. This doctrine of immobilisation was the result of expert opinion and limited case studies from the 1950s and 1960s and has remained relatively unchanged since its introduction (Farrington, 1967; Myer & Perina, 2016; Rogers, 1957). These widely accepted methods of cervical spine immobilisation described above were adopted worldwide and were relatively unchallenged until the publication of a controversial paper by Hauswald et al. (1998). The Hauswald paper findings were in stark contrast to the assumptions we have come to accept that traditional immobilisation reduces the risks of disability. As the literature has provided little empirical evidence into the benefits of traditional immobilisation and indicated the potential for negative consequences, Myer and Perina (2016) suggested we have created a 50-year culture of immobilising with no evidence of patient benefit that may potentially cause harm. This culture has perpetuated a fear of the consequences of not routinely immobilising with no real evidence to support this fear.

This article highlights a focus group study that was conducted to help support and influence the development of a clinical trial within the Northern Trauma Network (NTN). The Spinal Motion Restriction Feasibility (SMRF) study will compare outcomes between adult trauma patients assessed as potential SCI randomised to either immobilisation with a semi-rigid collar or without a semi-rigid collar. The focus groups, as part of the SMRF study, were funded by a small research grant from the North East Ambulance Service NHS Foundation Trust (NEAS).

### Aim

The purpose of this focus group study was to explore the views and perspectives of pre-hospital care providers about immobilising patients without using a semi-rigid collar for potential SCI.

## Methods

### Qualitative approach and research paradigm

Focus groups were chosen to allow individuals to discuss and comment on a research topic from a personal perspective (Powell et al., 1996). Focus groups allow researchers to capture thoughts, feelings and experiences, obtain several perspectives about the same topic and investigate collective understandings of a concept (Race et al., 1994). Although individual interviews were considered, the number of potential participants, limited resources, time restraints and practicality discounted this option. There is no consistency in the recommended numbers or length for focus groups within the literature, and a pragmatic approach to size and homogeneity was undertaken by the research team as discussed by Freeman (2007). In the context of this project many participants wished to attend, of which the majority were paramedics and all were from a single trauma network. The relatively large groups at each focus group (n = 15) were easily managed and data saturation occurred within 60 minutes, although more time was available.

An informal organic approach to the focus group discussion was adopted to minimise interruption and allow conversations to take their natural course. To help guide conversations that may be hesitant, various prompts were used to simulate the group discussions:

- What do you think of the new spinal motion restriction (not using semi-rigid collars) protocol?
- Would you feel confident managing potential SCI patients without a semi-rigid collar?
- What would you change about the protocol (if anything)?
- Can you identify any risks to patients?
- Can you identify any risks to staff?
- How can the organisation / clinical team support staff during the new practice of immobilising without using a semi-rigid collar?

In practice, the prompts were helpful but conversations naturally highlighted the topics and therefore they were not always needed.

During transcription, participants were given codes to provide basic information to support the analysis process. Within this article, direct quotes are simply attributed to participants alphabetically, so as to not identify any individual. The original intention was to use the terms ‘clinician’ and ‘non-clinician’ but all quotes used in the final paper originated from NHS clinicians.

### Researcher characteristics and reflexivity

LT and GS conducted the focus groups. Both are experienced specialist paramedics for trauma and familiar with the research topic but not experienced with focus group research. As such, practice sessions with more experienced colleagues and continuous reflection and peer debriefing with the research team were undertaken throughout the project. Variance in participant experience, profession, education and history suggested that the semi-structured format would be suitable to explore complex issues and provide the reflexivity to probe for further information or clarification (Bryman, 2016). There were naturally quieter members within each group. It was made clear in the introductions that everyone’s view should be heard, to aid transcription as well as ensuring every participant who wished to contribute could do so. Having an observational facilitator to highlight any issues minimised the risk of single participants dominating group dynamics and biasing the views of others within the group. This facilitator also highlighted when a quieter participant indicated they wished to contribute.

### Context

NEAS is the main ambulance service provider within the NTN and covers 8365 km^2^ with a mixture of remote rural geography and densely populated cities and towns. The NTN comprises two major trauma centres, eight trauma units and two Helicopter Emergency Medical Service bases as well as co-responders such as voluntary ambulance services, fire and rescue, police and community volunteers.

### Sampling strategy

A purposive sample of pre-hospital trauma care providers were selected for their experience in the pre-hospital setting. The participants were recruited by virtue of their membership of a pre-hospital trauma workshop group, with members originating from different sources (see Table 1) and organised throughout the NTN region at separate locations to ensure that any potential local idiosyncrasies were accounted for (such as rural/urban, differences in co-responding services, e.g. different police and fire and rescue services).

**Table 1.** Focus group participants.

Role	Focus group 1	Focus group 2	Focus group 3	Total
Paramedic (NHS)	13	11	9	33
Ambulance technician/care assistant (NHS)	2	2	–	4
Ambulance technician (non-NHS)	–	–	2	2
Police	–	–	2	2
Firefighter	–	2	–	2
Emergency dispatcher / call taker	–	–	2	2
**Total**	**15**	**15**	**15**	**45**

### Data collection methods

Two electronic dictation devices were used to record the sessions. Digital recordings of the conversations were transcribed verbatim and anonymised using alpha numeric codes prior to coding and analysis. GS compiled notes throughout the focus groups while LT chaired the sessions.

### Data analysis

Thematic analysis of data commenced after the first focus group and constant comparison was utilised following the Pope et al. (2000) framework. The method described by Saldana (2013) was used for coding to identify any emerging conceptual or theoretical themes.

NVivo qualitative data analysis software (QSR International Pty Ltd., Version 11, 2015) was used to manage and explore the data.

## Results

Three focus groups were conducted between February and March 2018, with 15 participants in each. Participants were all exposed to patients sustaining trauma within their professional roles and worked within the NTN region (Table 1). Six intertwined themes were identified during the discussions (communication, conflict, education/training, empowerment, risk and the patient), with many factors coded into multiple themes as they were so intrinsically linked. The themes, sub-themes and factors are summarised in Table 2.

**Table 2.** Themes, sub-themes and factors identified with regards to immobilising without use of a semi-rigid collar.

**Communication**		**Empowerment**	
**Sub-theme**	**Factors**	**Sub-theme**	**Factors**
Perceptions	Patient, public and inter-professional opinions which are often at conflict	Reassurance	Staff are protected Clinical governance Education and training Previous negative experiences of using rigid collars
	The need for public awareness		
	The positive and negative influence of all media formats		
Collaboration	Need to liaise with all emergency service providers, first responders and hospital staff	Ownership	Pre-hospital clinician-led project Positive confidence Bespoke care
	Need for joint working/education		
**Conflict**		**Risk**	
**Sub-theme**	**Factors**	**Sub-theme**	**Factors**
Professional	On scene (other emergency services)	Insurance claims	‘Whip-cash’ culture
	At hospital (viable dependent on hospital)		Medical negligence
Public	Perceptions (media)	Patient injury	From non-collar use
	‘Whip-cash’ culture (medical negligence)		From collar use
		Fear	Litigation
**Education/training**		**The patient**	
**Sub-theme**	**Factors**	**Sub-theme**	**Factors**
Public	Public awareness	Patient-centred care	Bespoke for the situation
	Media (all formats)		Reduced movement
	Promoting the results of the trial (positive/negative)		Reduced pain
Patients	On scene		Reducing anxiety
	At hospital	Consent	Specific to the collar trial
Professionals	Internal (NEAS)	Exclusions	Specific to the collar trial
	External (NTN): hospital, other emergency services		Unconscious/paralysed patients
	Equipment (standardised)	Complications	Agitation
	Joint training and collaboration		Anxiety
	Empowering staff with the knowledge of current research and clinical practice		Non-tolerating
			Intoxication
			Anatomical challenges (kyphosis)

### Communication

There were two sub-themes within the theme of communication: perceptions and collaboration (Table 2).

A significant issue highlighted at all focus groups was that of perception from various agencies and the public regarding immobilisation practices, as the traditional method of immobilisation is so ingrained into our popular culture.

Participant A: *This is what we’ve always done.*

It was widely recognised that our biggest collaborators in immobilisation practices are the fire and rescue services. It was reassuring that relationships and collaboration have improved in the region and our fire and rescue colleagues are highly skilled and aware of new clinical practices (largely due to local joint training and education programmes).

Participant B: *[we] do a lot of co-responding and they [now have] more training … more willing to listen, we’ve got a better relationship.*

The sub-theme of collaboration was intricately linked to the issue of conflict and how to prevent it.

### Conflict

Although there was an awareness of improved collaboration and understanding with other emergency services, concerns about potential conflict with regards to immobilising patients without using a semi-rigid collar still remained within very specific areas of our region.

Participant C: *there is a potential of opening a full can of worms with the Fire Brigade.*

This concern was also raised with regards to conflict with professional staff on arrival at local hospitals and collar/non-collar use.

Participant D: *there’s the patient and public perception of what we’re going to do and there’s also the hospital perception.*

Participants highlighted cases where conflict had occurred at hospital because pre-hospital clinicians had fitted a semi-rigid collar.

Participant E (highlighting the comments at patient handover):* What have you got the collar on for? … you shouldn’t be using collars.*

This theme reflected the variation in reception at different hospitals within the region. Specialist hospitals were more accepting of non-collar use, whereas smaller non-specialist hospitals, who are not as exposed to patients sustaining trauma, had the opposite attitude to collar or non-collar use.

An area of potential conflict with fire and rescue services was widely acknowledged to be mitigated by working in partnership with emergency service colleagues by providing information and support through collaborative training and education.

### Education/training

The education and training theme (linked to communication and preventing conflict) had three sub-themes that related to clinical staff and the patient as well as the wider public (Table 2).

Participant F: *… quite keen to have … a campaign … public awareness, where it’s put out on the radio, the local news or something, just saying this is what we’re going to do and this is why we’re doing it.*

Most participants regularly work with other emergency services and acknowledged that regular collaborative training and standardised equipment have improved working relationships and confidence.

### Empowerment

There was a distinct feeling by participants that they were empowered to provide bespoke patient-centred care. For many it felt wrong to apply a semi-rigid collar that was causing discomfort. Empowering staff to make a clinical assessment and base their management on the patient’s needs was acknowledged by the participants as a common-sense approach.

Participant G: *it is about providing that bespoke care to that individual person at that moment in time and how they present to you.*

There was also a perception that influencing potential change in practice from a grassroots approach by helping to support the design of (and participating in) clinical research was empowering and promoted confidence in the study.

Participant H: *it’s empowering … as clinicians … to make those decisions.*

Parallel to the feelings of empowerment was the perception that there are also potential risks when using and not using semi-rigid collars.

### Risk

Although participants supported moving away from routine semi-rigid collar use, there was a concern with regards to the risks of litigation from non-collar use.

Participant I: *another difficulty is [medical negligence], you’re going to have … lots of people expecting that they’re going to end up with X amount of money for an accident.*

The groups all highlighted the emergence of the medical negligence culture of insurance companies pursuing claims arising from an accident. There were several discussions about the potential for perceived medical negligence from non-collar use and the expectation of care perpetuated in contemporary media. This was linked to public education and communication and was a common theme, leaving many participants anxious about what would be a significant change from current practice.

Participant J: *a lot of us would like as a common-sense approach [to collars] but with the reassurance … that [the organisation] is not going to sack you.*

In addition to risk from non-collar use in patients with reduced consciousness/paralysis it was also acknowledged there were risks in using a semi-rigid collar in other patient groups (increasing intracranial pressure, unnatural position of spine in kyphotic patients, etc.).

Participant K: *we could potentially be making these people worse [by using a semi-rigid collar].*

An insightful comment by one participant concerned the risk of complacency in relation to not using a semi-rigid collar and potentially forgetting to manage the spine and other injuries adequately.

Participant L: *there is always a danger when you roll out any kind of treatment you can become complacent in relation to other treatments. Like you wouldn’t fully immobilise them, you might then forget to put a pelvic binder on.*

Although the issues of risk were a real concern, the belief that patient-centred care would be a fundamental aspect of immobilisation options was central within all discussions.

### The patient

Delivering patient-centred care was a focal point of all conversations, with anecdotal examples describing cases where patients had obvious increased agitation, pain, discomfort and anxiety when immobilised with semi-rigid collars.

Participant M: *why are they agitated? Have they got painful injuries? Can you give them some morphine? Can you reassure them?*

There was a widespread acknowledgement that patients expressed relief from these symptoms as soon as the collar was removed.

A few areas of discussion revolved around specific issues with consenting and excluding participants from the SMRF (non-collar) study, which included the complexities of patients who were unconscious or paralysed and/or could not self-immobilise. Consequently, this will influence how the study is designed.

Another significant issue related to non-cooperative, agitated or intoxicated patients. The consensus suggested these groups would be challenging to immobilise regardless and attempting to use a semi-rigid collar may cause unnecessary movement/pressure on the spine. This was also true for patients with difficult anatomy such as a kyphotic spine.

Participant N: *I suppose drink and drugs isn’t going to matter because … they’re going to be agitated and [not] complying anyway.*

There was a general acknowledgement that, for appropriate patients, the move away from routine collar use was a positive step in patient care.

## Discussion

### Key results

The main themes are intrinsically linked, and their complex interactions make it difficult to draw comparisons from literature that supports immobilisation and semi-rigid collar use.

As the literature has provided little empirical evidence into the benefits of traditional immobilisation and indicated the potential for negative consequences, Myer and Perina (2016) suggested we have created a 50-year culture of immobilising with no evidence of patient benefit that may potentially cause harm. Immobilisation practices are so ingrained within our professional culture they are part of established trauma education. In 1976, the principles of advanced trauma life support (ATLS) were conceived by Dr James Styner. The first ATLS course was held in 1978 and ATLS was adopted by the American College of Surgeons in 1980. ATLS remains part of the syllabus of most emergency departments and pre-hospital care providers (including the Royal College of Emergency Medicine’s curriculum) (American College of Surgeons, 2012).

The widely accepted methods of spine immobilisation described above were adopted worldwide and relatively unchallenged until the publication of the controversial paper by Hauswald et al. (1998). The Hauswald paper’s findings were in stark contrast to the assumptions we have come to accept that traditional immobilisation reduces the risks of disability.

The overall feeling within the groups with regards to adopting a non-collar approach to immobilisation was positive, with a pragmatic and realist attitude towards a potential change to future practice. It was acknowledged that limited and dated literature that initiated immobilisation practices has already been challenged by established national bodies. The consensus statement from the faculty of pre-hospital care (Connor et al., 2013) highlighted growing concerns for traditional immobilisation and adopted the pragmatic view of applying a clinical assessment to ensure bespoke patient-centred care. Although the National Institute for Health and Care Excellence (2016) trauma guidelines recommend the use of collar, scoop, stretcher, blocks and tape, there is a caveat that anatomy, deformity, confusion or agitation requires a pragmatic approach and to aim for a position that is comfortable for the patient.

Other pre-hospital care providers have already changed their clinical practice and removed routine use of semi-rigid collars for immobilisation. Kornhall et al. (2017) highlight the systematic review commissioned by the Norwegian National Competence Service for Traumatology who recommended that, while SCI is a rare event, potential spinal injuries should still continue to have spinal stabilisation. The consensus was not to abandon a strategy of immobilisation but to adopt a selective approach with minimal handling to reduce spinal movement, reduce pain and potentially promote haemostasis. However, spinal stabilisation should ‘never delay or preclude lifesaving interventions’ (Kornhall et al., 2017).

The Quinn and Enraght-Moony (2015) review added credibility to the Queensland Ambulance Service approach to immobilisation. Their new approach to spinal motion restriction practices not only removed the routine use of semi-rigid collars but adopted a more selective approach to spinal motion restriction and recognised the futility of immobilising isolated penetrating trauma. Spinal motion studies using healthy volunteers or cadavers provide conflicting evidence when studying spinal movement under various conditions. Perry et al. (1999) identified that various techniques/equipment routinely used in traditional immobilisation do not eliminate neck motion during extrication or transport. Other studies have identified that using conventional techniques and equipment may actually increase neck motion (Dixon et al., 2014; Engsberg et al., 2013).

Dunham et al. (2008) identified the association of increased intra-cranial pressure when using a semi-rigid collar. The 2001 Cochrane review highlighted that the use of cervical collars can also contribute to airway compromise and may also contribute to the increased risk of mortality and morbidity (Kwan et al., 2001).

Oteir et al. (2014) stated that the most important variables in determining improved outcomes for spinal injured patients were effective resuscitation and the early transfer of patients to specialist spinal care units.

### Limitations

Participants were all members of a trauma workshop and potential volunteers for the SMRF study and therefore a self-selecting group, which may bias their views on current immobilisation practices. Non-NEAS clinical staff were all potential stakeholders within the SMRF study. However, NEAS paramedics were the primary participants within the focus groups and may not reflect the wider pre-hospital perspectives of non-collar use. The participants all worked within a single trauma network and their views potentially differ from other regions or nations; the concepts should be generalisable and transferable to other trauma networks/systems.

Focus group discussions present the participants’ views of reality and there may be different understandings and perspectives between researcher and participant. Within the context of this research, the lead author is an experienced paramedic working in the same trauma system following the same guidelines, which should minimise misinterpretation of the data. Initial transcription and original coding and interpretation were cross-checked by another experienced paramedic who was present during all focus groups. However, as specialist paramedics, the researchers who facilitated the focus groups may have unintentionally biased the content and direction of the discussions. Participant checking of the original transcripts for accuracy was also completed.

## Conclusions

This focus group study explored the views and perspectives of pre-hospital care providers immobilising patients without the use of a semi-rigid collar for potential SCI. The consensus of the participants supported a pragmatic approach to managing potential SCI that provides safe, high-quality patient-centred care, therefore supporting the SMRF study concept.

## Acknowledgements

The researchers acknowledge the contribution of Wilma Harvey-Reid for her valuable time in proofreading and manuscript layout.

## Author contributions

LT: Lead author, focus group facilitator, data collection, analysis and interpretation of data/literature.

GS: Contributing author, focus group facilitator, peer discussion, analysis and proofreading.

CB: Principal investigator, contributing author, focus group information and interpretation of data.

CH: Principal investigator, contributing author, focus group information and interpretation of data.

GM: Contributing author, mentor, analysis and interpretation of data/literature.

PM: Contributing author, mentor, analysis and interpretation of data/literature.

All authors have read and approved the final manuscript.

LT acts as the guarantor for this article.

## Conflict of interest

LT: Chief investigator on Spinal Motion Restriction Feasibility (rigid collar) Study.

GS, CB and CH: Principal investigators on Spinal Motion Restriction Feasibility (rigid collar) Study.

GM: Editor of the BPJ.

## Ethics

The protocol for the study was reviewed and approved by Northumbria University Ethics Committee (ref. 5714). Each participant received a participant information sheet prior to the focus groups and consented on the day. The participants were recruited by virtue of their membership of a pre-hospital trauma workshop group, with members originating from different sources, and not because they were members of an NHS institution; therefore, this study did not require HRA approval.

## Funding

Small research grant, North East Ambulance Service NHS Foundation Trust.
